# Refractive Index-Based Terahertz Sensor Using Graphene for Material Characterization

**DOI:** 10.3390/s21238151

**Published:** 2021-12-06

**Authors:** Aruna Veeraselvam, Gulam Nabi Alsath Mohammed, Kirubaveni Savarimuthu, Jaume Anguera, Jessica Constance Paul, Ram Kumar Krishnan

**Affiliations:** 1 Department of ECE, Sri Sivasubramaniya Nadar College of Engineering, Chennai 603110, India; arunav@ssn.edu.in (A.V.); gulamnabialsathm@ssn.edu.in (G.N.A.M.); kirubavenis@ssn.edu.in (K.S.); jessica17067@ece.ssn.edu.in (J.C.P.); ram17127@ece.ssn.edu.in (R.K.K.); 2 Department of ECE, Universitat Ramon Llull, 08028 Barcelona, Spain

**Keywords:** THz sensors, metamaterials, graphene, sensing, THz imaging

## Abstract

In this paper, a graphene-based THz metamaterial has been designed and characterized for use in sensing various refractive index profiles. The proposed single-band THz sensor was constructed using a graphene-metal hybridized periodic metamaterial wherein the unit cell had a footprint of 1.395λ_eff_ × 1.395λ_eff_ and resonated at 4.4754 THz. The realized peak absorption was 98.88% at 4.4754 THz. The sensitivity of the proposed metamaterial sensor was estimated using the absorption characteristics of the unit cell. The performance of the sensor was analyzed under two different categories, viz. the random dielectric loading and chemical analytes, based on the refractive index. The proposed THz sensor offered a peak sensitivity of 22.75 GHz/Refractive Index Unit (RIU) for the various sample loadings. In addition, the effect of the sample thickness on the sensor performance was analyzed and the results were presented. From the results, it can be inferred that the proposed metamaterial THz sensor that was based on a refractive index is suitable for THz sensing applications.

## 1. Introduction

Terahertz (THz) radiation lies between the microwave and infrared frequencies. The term THz signifies a trillion cycles per second. Recently, the research into THz radiation has attracted interest due to its non-ionizing nature and high penetration depth. It is capable of penetrating through materials that do not support the propagation of the electromagnetic spectrum outside of the THz region. The research on this portion of the spectrum remains relatively unexplored when compared to the research on well-developed technology, such as microwave, optical and x-rays. THz radiation has been widely applied in communication, spectroscopy, imaging, sensing and material characterization. Unlike UV radiation or X-rays, THz radiation has low photon energy and does not change the chemical structure of the material that it is applied to [1]. Due to its highly penetrative and non-destructive nature, it is widely used in different applications. The THz frequency ranges from 0.1 to 10 THz. This range is widely used due to its penetration capability with low energy consumption. Additionally, THz is used in other applications such as the detection of explosives in security applications [2], high-precision monitoring in pharmaceutical production [3], non-destructive and non-contact testing [4] and the detection of blood components [5].

THz devices are predominantly designed using metamaterials. A metamaterial is an artificial electromagnetic material that is composed of a periodic array of unit cells. Metamaterial devices possess the ability to control and manipulate the electromagnetic waves incident on them at sub-wavelength dimensions [6]. They are especially commonly used at THz frequencies due to their strong absorption properties. A perfect metamaterial absorber, which was designed using a split ring resonator on the FR4 substrate, was realized by Landy et.al. in [7]. In [8], the authors had designed an ultra-sensitive centrosymmetric double F-shaped metal resonator, using Teflon as the dielectric material, which operated at 5.92 THz. A tri-band metamaterial absorber was presented in [9]. In [9], symmetrical split rings had been designed on a polyimide substrate that resonated at 0.325 THz, 0.63 THz and 1.07 THz. Similarly, in [10], a four-band THz metamaterial absorber was designed using square ring resonators on the dielectric layer whose ε = 4.1 and which resonated at 0.77, 1.13, 1.53 and 2.06 THz. A five-band nested split ring resonator was reported in [11], the unit cell size of which was 60 µm × 60 µm on a dielectric with ε = 3. An average peak absorption of 99% was obtained between 0.5 to 3 THz. In [12], the sensor was constructed using a dielectric waveguide which used an anti-resonant reflecting layer that formed multiple resonant peaks. It resonated at 0.15 THz with a sensitivity of 22.2 GHz/RIU. In [13], the authors proposed an oscillator-based sensor for detecting the dielectric materials using a capacitive metal mesh which acted as a reflector for the oscillators. This had a sensitivity of 2.6 GHz/RIU for distinguishing dielectric materials and resonated at 0.1 THz. A sensor that was based on photonic crystal slabs, which are extremely sensitive to variations in the environment, was presented in [14]. The sensor proposed in [14] was fabricated on a quartz substrate with a square lattice of air holes in a slab of silicon for detecting the refractive index which resonates at 0.508 THz with a sensitivity of 23.08 GHz/RIU. Thus, the investigation of different dielectric substrates has been analyzed in the literature for the development of metamaterial THz devices. In this research, the THz sensor was developed using a metamaterial which incorporated graphene as the conducting material. Graphene consists of a layer of carbon atoms that are arranged in a honeycomb fashion. Graphene is an interesting material in THz applications due to its atomic strength, easy tunability and high kinetic inductance [15]. The chemical potential of graphene can be adjusted externally through a suitable DC bias network [16]. Similarly, another parameter for tuning the characteristics of a graphene-based sensor is the dielectric constant of the graphene, which is modelled as a thin layer using Equation (1) [16], given below. By adjusting the chemical potential, the conductivity of graphene can be adjusted, which, in turn, is reflected on the permittivity of the material under investigation.

(1) $$\varepsilon =1+\frac{i\sigma }{\varepsilon_{0\;}\omega t}$$

where $\varepsilon_{0\;}$is the vacuum permittivity with the layer thickness denoted by t. In line with this, the researchers who presented [16] have constructed an array of one rectangle-based and two triangle-based THz absorbers in order to obtain resonances at 6.62 THz and 9.36 THz with an average absorptivity of 99.4%. In [17], a graphene-based THz absorber using a dual-ring structure was designed with a footprint of 34 µm × 34 µm. The absorber designed in [17] resonated at 1.6 THz and 2.89 THz for the process of tuning the chemical potential to 0.2 eV.

With reference to applications that are based on a chemical analyte, THz devices are used for the label-free diagnosis of malignant tumors, detection of blood composition and so on. The proposed THz sensor can be used extensively to sense a material based on the variations in its refractive indices. Some researchers have attempted a theoretical study on the development of THz sensors using metamaterials. In [18], a fan-shaped THz sensor was designed to resonate at 4.87 THz for detecting the refractive index of an analyte with an absorptivity level of 99.6%. In [19], the authors reported a multiband THz sensor that used concentric square and octagonal loops, the absorptivity of which was 99%. 

In this paper, a graphene-metal hybrid THz sensor was designed and its ability to detect refractive index variations was theoretically demonstrated. The proposed THz sensor had an estimated absorptivity of 98.88% and showed a peak sensitivity of 22.75 GHz/RIU. The rest of the present manuscript is organized as follows: Section II presents the design, evolution and analysis of the THz sensor. Section III describes the estimation of sensitivity for various materials and analytes, based on the refractive index. Section IV presents the conclusion.

## 2. Sensor Design

### 2.1. Construction

**Figure 1:**
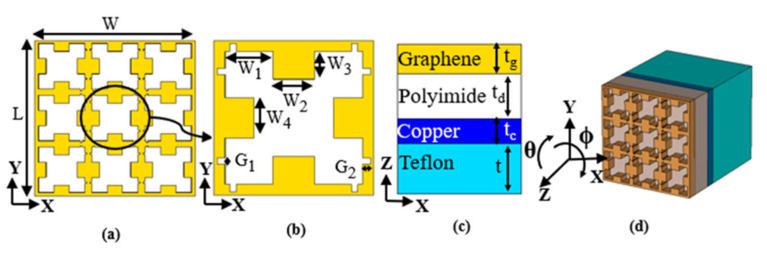
Structure of the THz sensor (**a**) Top view (**b**) Top view of unit cell (**c**) Side view (**d**) Perspective view.

The geometry of the proposed metamaterial unit cell for THz sensing is illustrated in Figure 1. The optimized dimensions of the proposed THz sensor are given in Table 1. The overall footprint of the proposed THz metamaterial sensor was 50(L) × 50(W) µm. The top view of the proposed THz sensor is shown in Figure 1a. The magnified view of the graphene patterning is described in Figure 1b. The side and perspective views of the designed sensor are described in Figure 1c,d, respectively. The patterned graphene layer that was located on the top had a series of interconnected square-shaped resonators along the horizontal and vertical planes of the unit cell. Among the various conductive materials that can be used for sensor development, graphene is an attractive choice due to its chemical, electrical and mechanical properties. The Fermi level of graphene can be adjusted in order to obtain a peak absorptive wavelength [20] as per the user’s requirement. In addition, graphene has the potential to excite surface plasmon effects [21]. The surface conductivity of graphene can be estimated using Kubo’s formula [22], which includes inter-band and intra-band parts, such as:

(2) $$\sigma \left(\omega \;,\;E_{f},\;\Gamma \;,\;T\right)=\sigma_{int\;er}+\sigma_{int\;ra}$$

(3) $$\sigma_{int\;ra}=\frac{2k_{B\;}T_{e}{}^{2}}{\pi h^{2}}\ln\left(2\;\cos h\;\frac{E_{f}}{2k_{B\;}T}\right)\;\frac{i}{\left(\omega +i\Gamma \right)}=\frac{\alpha }{-i\omega +\Gamma }$$

(4) $$\sigma_{int\;er}=\frac{e^{2}}{4h}\;\left[H\left(\frac{\omega }{2}\right)+i\;\frac{4\omega }{\pi }\;\int_{0}^{\infty }\frac{H\left(\Omega \right)-H\left(\frac{\omega }{2}\right)}{\omega^{2}-4\Omega^{2}}\;d\Omega \right]$$

(5) $$H\left(\Omega \right)=\sin h\left(\frac{h\Omega }{k_{B\;}T}\right)/\left[\cos h\;\left(\frac{h\Omega }{k_{B\;}T}\right)+\cos h\;\left(\frac{E_{f}}{k_{B\;}T}\right)\right]\;$$

where ω is the angular frequency, $E_{f}\;$is the Fermi energy of the graphene, Γ is the collision angular frequency, T is the temperature, $k_{B\;}$ is the Boltzmann constant, e is the elementary charge and h is the reduced Planck’s constant. Graphene’s conductivity is higher when its Fermi energy is greater than half of its photon energy (E_f_ > hω/2), at this point the inter-band part becomes negligible compared to the intra-band part, due to Pauli blocking. Thus, the conductivity of graphene is predominantly mediated by the intra-band effects.

The proposed sensor had a four-layer configuration, as shown in Figure 1c. The layer of the patterned graphene was synthesized on a polyimide substrate, the other side of which was coated with a copper layer. The copper layer beneath the polyimide acted as the ground layer. The entire sensor was developed over a polytetrafluoroethylene (Teflon) base in order to achieve the necessary level of mechanical stability. The electromagnetic (EM) energy that was incident on the sensor was able to be configured in order to achieve the desired absorption, reflection and transmission properties. In this research, the THz sensor was configured as an electromagnetic absorber with a narrow absorption spectrum. The sensor modelling was carried out using CST Microwave Studio’s finite integration technique. Floquet mode theory, with periodic boundary conditions along the ‘x’ and ‘y’ directions and excitation along the ‘Z’ direction, was used to characterize the metamaterial unit cell. The absorptivity of the sensor was estimated using Equation (6) [19]:

A(ω) = 1 − R(ω) − T(ω) = 1 − |S_11_|^2^ − |S_21_|^2^ (6)

where A is the absorptivity or absorption coefficient and R and T are the reflectance and transmittance, respectively. In general, a perfect sensor has near-perfect absorption (i.e., unity). The perfect absorption is achieved only if the EM transmission is fully blocked by the sensor. Therefore, the transmission of the EM wave was prevented by the Fabry–Perot cavity [23] that was formed between the dielectric and ground layers. Thus, Equation (6) can be reduced to Equation (7) as given below.

A(ω) = 1 − R(ω) = 1 − |S_11_|^2^ (7)

**Figure 2:**
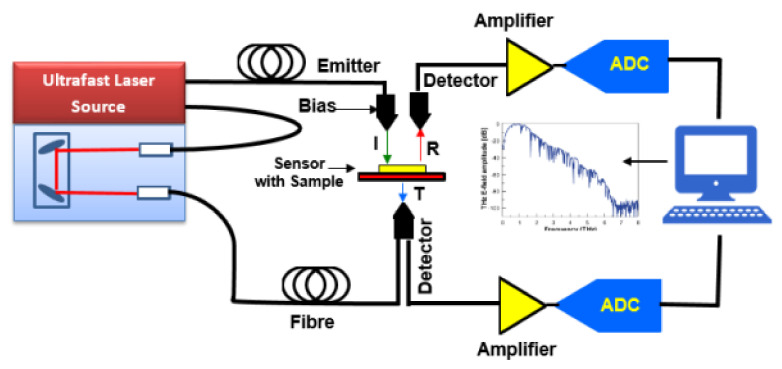
Schematic diagram of the working set up.

The schematic diagram of the working set up using the THz sensor is shown in Figure 2. The set up consisted of an ultrafast LASER source (THz radiation) which was focused and coupled to an emitter consisting of photoconductive antennas. Within the diagram, the antenna that was radiating the THz waves is labeled ‘Emitter’. The detector was positioned at a certain distance in order to enable the loading of the analytes that were to be tested. The THz waves incident (I) on the sample were reflected (R) and transmitted (T) and then collected using the detectors that were placed at appropriate distances. The reflected and the transmitted THz radiation was amplified and converted into digital signals which were then fed to a personal computer for signal processing. The processed signal provided information about the spectra of the reflected and transmitted signals. It is to be noted that the amount of reflection and transmission depended on the analyte that was loaded between the emitter and the detectors. The absorption characteristics of the sensor were considered in analyzing the characteristics of the analyte.

### 2.2. Evolution

**Figure 3:**
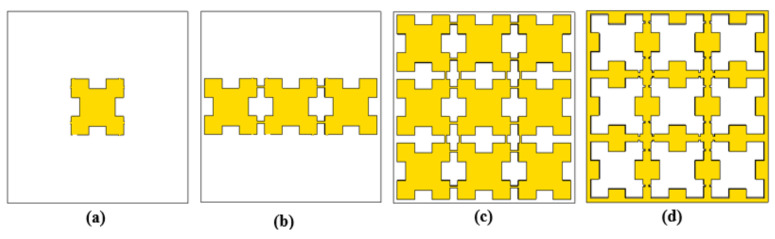
Evolution of the THz sensor: (**a**) Resonator 1 (**b**) Resonator 2 (**c**) Resonator 3 (**d**) Proposed Resonator.

**Figure 4:**
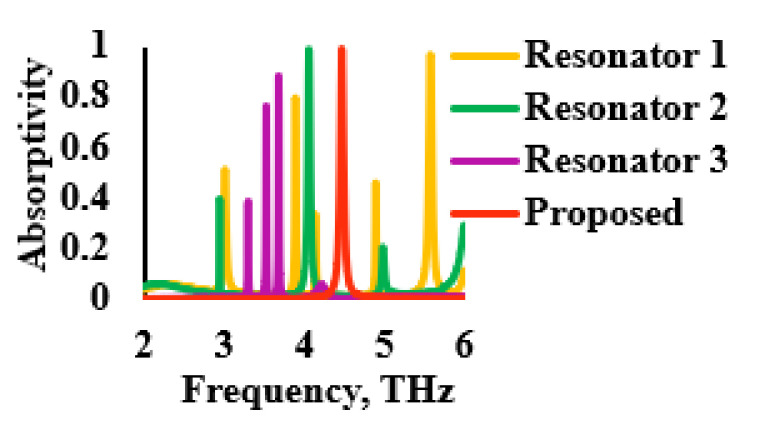
Absorption characteristics during different stages of evolution.

**Figure 5:**
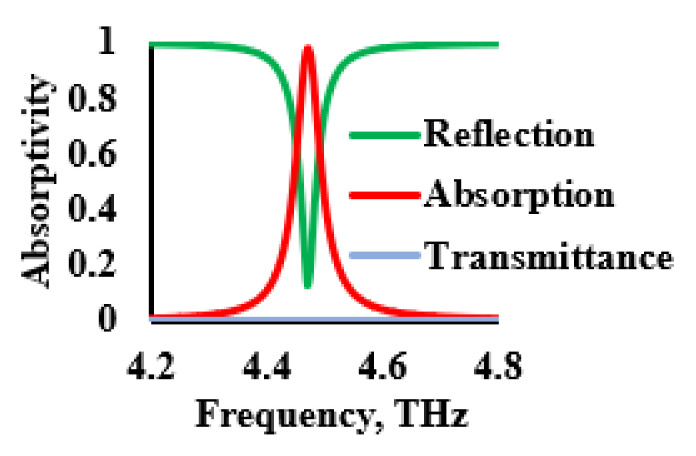
Reflection, transmittance, and absorption characteristics of the THz sensor.

Figure 3 shows the evolution of the proposed THz sensor and Figure 4 describes the associated absorption characteristics that were found during the evolution stages of the proposed sensor. The evolution of the sensor began with a patch that was implemented on a polyimide substrate, referred as resonator 1, as shown in Figure 3a. The patch had multiple narrow absorption peaks, as shown in Figure 4. Thus, in order to improve and stabilize the absorption, the unit cell was replicated, as described in Figure 3b. This modification reduced the number of resonances, as illustrated in Figure 4. The number of unit cells was increased, as shown in Figure 3c, in order to further reduce the number of resonances. However, an optimum absorption characteristic was not realized due to the high level of reflectance. Thus, to improve the absorptivity, a periodic patch resonator surface, as shown in Figure 3d, was designed by complementing the previous stage of evolution. This provided good absorptivity and stability since this stage had resulted in the reduction of the amount of conductive portions of the sensor’s surface. Therefore, the final resonator had a conducting layer that consisted of a square-shaped periodic structure. The reflection, transmittance and absorption characteristics of the proposed THz sensor are shown in Figure 5. It was inferred from the data that are shown in this figure that the transmittance and reflection were minimal, with a high absorptivity of 98.88% at 4.4754 THz.

**Figure 6:**
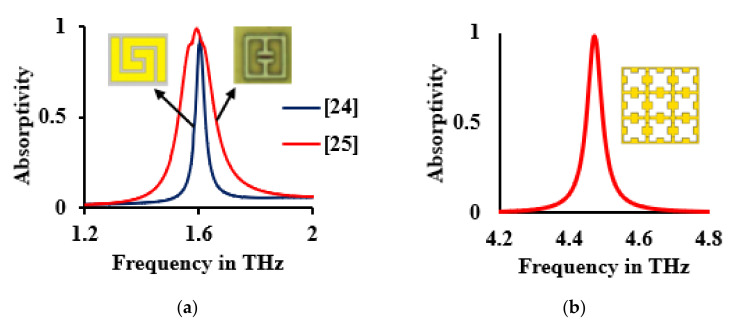
Absorption characteristics of (**a**) Spiral absorber and split ring resonator, (**b**) Proposed sensor.

In addition to this, the accuracy of the simulation was verified by repeating the designs that were proposed in [24,25]. During this phase, the simulation setup could successfully reproduce the results reported in [24,25], ensuring the accuracy of the theoretical analysis. This accuracy was met by fixing the number of mesh lines per wavelength at 15 and the accuracy at 10^−6^ in the frequency domain solver of the full-wave EM solver in CST Microwave Studio. Figure 6 shows the absorption characteristics of the absorbers that were reported in [24,25], as well as those of the proposed sensor. Thus, it can be guaranteed that the present sensor, if fabricated, could produce identical results to those that were demonstrated in [25].

**Figure 7:**
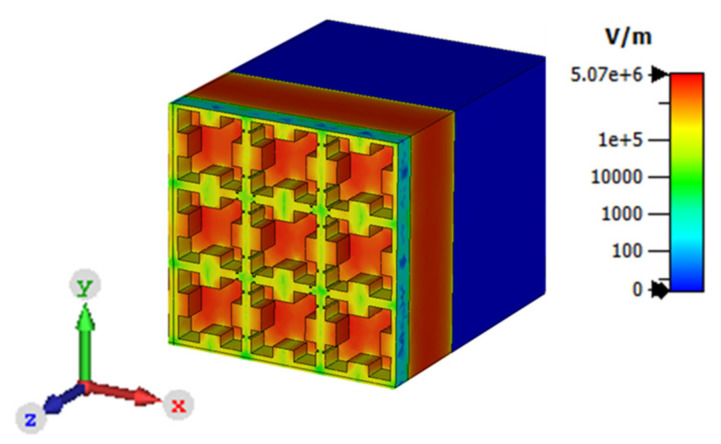
The magnitude of the E-field intensity at 4.4754 THz.

**Figure 8:**
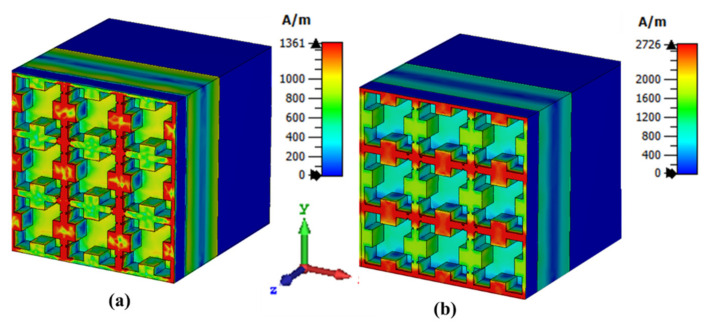
Surface plasmon density at 4.4754 THz (**a**) TE mode (**b**) TM mode.

Furthermore, the operation of the proposed sensor was validated by using electric field intensity and surface current density plots. The E-field distribution of the metamaterial THz sensor at 4.4754 THz is shown in Figure 7. From the figure, it is evident that the proposed sensor exhibited a propagative surface plasmon effect. The confinement of electrons in the proposed sensor is shown in Figure 7. Based on Coulomb’s effect, the oscillating frequency of the electrons enhanced the electric field on the particle’s surface. Similarly, the plasmon-induced transparency (PIT) was used in order to confine the electromagnetic fields which provided a path to achieve highly sensitive sensors [23]. The surface plasmon density at 4.4754 THz for the TE and TM modes is shown in Figure 8. It was inferred, from Figure 8a, that the plasmon density was high along the vertical axes of the square lattice at ϕ = 0°. Similarly, at ϕ = 90°, the plasmon density was concentrated along the horizontal axes of the square lattice, as shown in Figure 8b.

### 2.3. Influence of External Bias 

**Figure 9:**
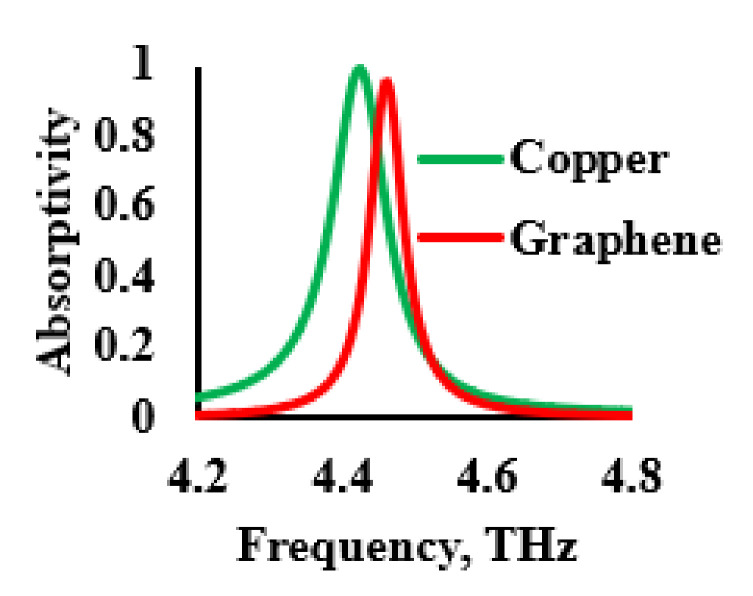
Absorption characteristics of different conductors used in the development of sensor.

In this section, the performance of the THz sensor for different conductors is analyzed. Copper with frequency-independent conductivity of 5.8 × 10^7^ S/m, for use along with graphene, was considered for its unique property of tunability. Figure 9 shows the influence of the conductors on the absorption characteristics. It was inferred from Figure 9 that the usage of copper led to higher absorptivity with a wide bandwidth. In order to improve the sensitivity of the sensor, narrow absorption peaks were preferred. The graphene had a slightly lower value of absorption but a very narrow peak.

**Figure 10:**
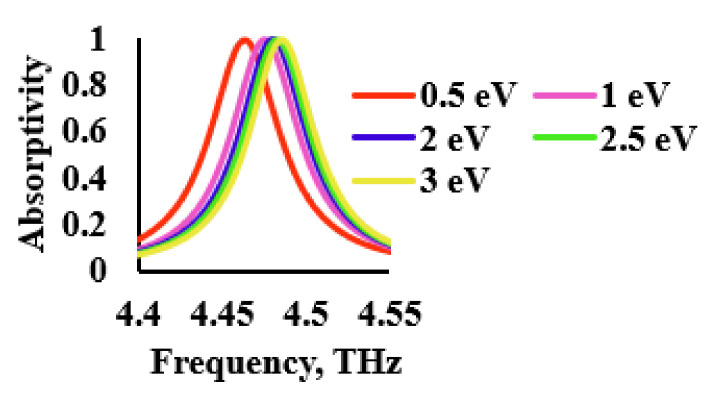
Sensor performance for varying graphene chemical potentials.

The absorption frequency of the graphene-based THz sensor could be controlled using the external voltage bias (V_DC_), which altered the Fermi level of the graphene material. The Fermi energy (E_f_) of the graphene was related to the V_DC_ using Equation (8) [21]:

(8) $$\left|E_{f}\right|=hv_{f}\sqrt{\frac{\pi \varepsilon_{r}\varepsilon_{0}V_{DC}}{et_{s}}}$$

where $\varepsilon_{r\;}$is the permittivity of the spacer, $\varepsilon_{0}$ is the free space permittivity, $t_{s}\;$is the thickness of the spacer, $V_{DC}$ is the dc voltage bias and $v_{f}$ is the Fermi velocity (1.1 × 10^6^ m/s). Equation (8) was used to calculate the required voltage bias for the change in Fermi energy. Therefore, when the external potential was varied, the chemical potential of the graphene could be altered, which led to an average shift in frequency of 10 GHz/eV, as shown in Figure 10.

## 3. Sensitivity Estimation

### 3.1. Effect of Varying Dielectric Constant

**Figure 11:**
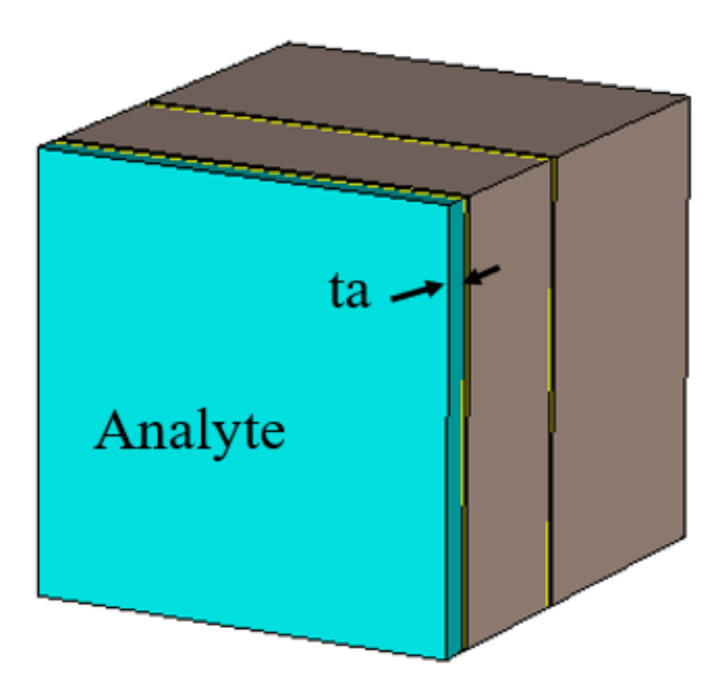
Experimental setup for sensitivity estimation.

As reported in this section, the sensitivity of the THz sensor was estimated by loading the sensor with different materials with varying dielectric constants. Each sample was characterized by its unique refractive index profile. The simulation setup of the THz sensor with analyte loading is described in Figure 11. The sensing mechanism of the proposed sensor can be analyzed using the perturbation theory and the equivalent medium theory. As per the perturbation theory, the relative change in resonant angular frequency with respect to the change in dielectric constant is described by Equation (9) [26]:

(9) $$\frac{\Delta \omega }{\omega_{0}}=\frac{-\int_{v_{0}}\left(\Delta \varepsilon {\left|\bar{E_{0}}\right|}^{2}+\Delta µ{\left|\bar{H_{0}}\right|}^{2}\right)dv}{\int_{v_{0}}\left(\varepsilon {\left|\bar{E_{0}}\right|}^{2}+µ{\left|\bar{H_{0}}\right|}^{2}\right)dv}=\frac{-\int_{v_{0}}\left(\Delta \varepsilon {\left|\bar{E_{0}}\right|}^{2}\right)dv}{\int_{v_{0}}\left(\varepsilon {\left|\bar{E_{0}}\right|}^{2}\right)dv}$$

where $\bar{E_{0}}$ and $H_{0}$ are the electric and magnetic field in the sensor without an analyte, respectively; and Δε and Δµ are the differential change in permittivity and permeability, respectively. From the equivalent medium theory, the effective dielectric constant of the loaded sensor was given by Equation (10) [26]:

(10) $$\varepsilon_{eff}=\varepsilon_{sub}+\alpha \;\varepsilon_{air}+\left(1-\alpha \right)\varepsilon_{r}$$

where $\varepsilon_{sub},$ $\varepsilon_{air}$, $\varepsilon_{r}$ are the permittivity of the substrate, air, and analyte, respectively, and α is the correlation coefficient between the air and sensor. The effective dielectric constant altered the effective capacitance of the THz metamaterial sensor. The effective capacitance (C_eff_) of the sensor depended on the device capacitance and sensing capacitance (C_sensor_). The device capacitance refers to the capacitance from the device with a dielectric constant sandwiched between the two metal layers. The sensing capacitance refers to the capacitance from the analyte that was loaded onto the device. Thus, the value of the C_sensor_ varies based on the refractive index (n) and thickness of the analyte. Thus, the change in sensing capacitance (C_sensor_) further varied the effective capacitance (C_eff_) of the metamaterial sensor [8]. Therefore, the change in C_eff_ affected the Q factor of the sensor, which is expressed as Q = 1/ωCR. In the proposed research, as the refractive index increased, so too did the effective capacitance, which reduced the Q factor. This, in turn, redshifted the resonant frequency of the metamaterial sensor.

**Figure 12:**
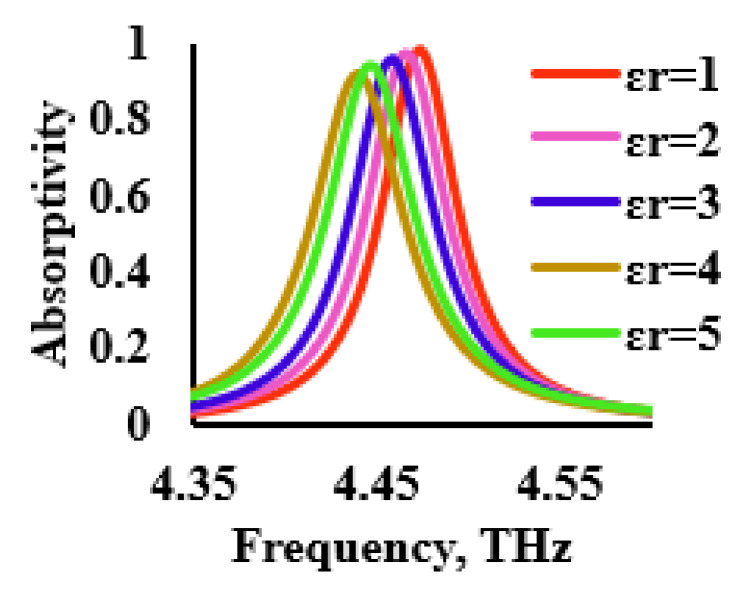
Performance of the THz sensor for various dielectric constants.

In line with the above discussion, the THz sensor was loaded with an analyte of varying dielectric constants, in the range of 1 to 5, with a sample thickness (t_a_) of 1 μm. The absorption characteristics of the analytes with different dielectric constants are shown in Figure 12. From Figure 12, it was inferred that a redshift of 10 GHz/Permittivity Unit (PU) was observed. The absorptivity decreased with the increase in the value of the dielectric constant. 

### 3.2. Effect of Varying Refractive Indices

**Figure 13:**
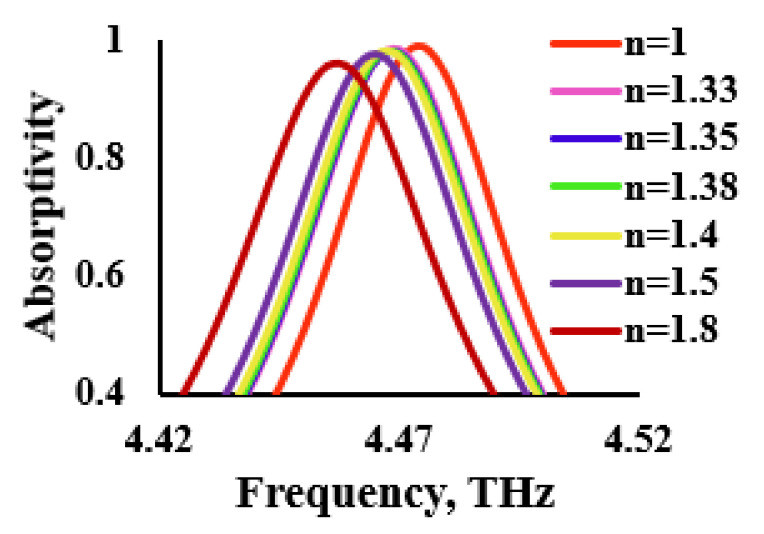
Sensor performance based on the refractive index.

This section presents the estimation of the proposed THz sensor for various analytes, based on their refractive index profiles. The refractive index of the analytes that were considered varies from 1.33 to 1.8 [27,28,29]. The designed THz sensor was loaded with analytes of different refractive indices for a constant thickness of 1 µm. The refractive index of the analyte was related to the dielectric constant using $n=\surd \varepsilon_{r}$. The absorption characteristics of the various refractive indices are shown in Figure 13. It can be inferred from this figure that the redshift occurred as the refractive index of the component increased, as is evidenced by the data that are provided in Table 2. It was also noted that the absorptivity was also gradually reduced with the increase in the refractive index.

The absorption characteristics were further investigated for frequency deviation, sensitivity, full width half maximum (FWHM), quality factor and figure of merit (FoM). Sensitivity was defined as the ratio of the frequency deviation (Δf) to the change in the refractive index (δn) whose mathematical expression can be given as S = Δf/δn. It was measured in terms of GHz/Refractive Index Unit (RIU). The FoM was defined as the ratio of sensitivity to FWHM, whereas the quality factor was defined as the ratio of resonant frequency to FWHM. The proposed THz sensor estimated a peak sensitivity of 22.75 GHz/RIU for the refractive index of 1.8 with a maximum frequency deviation of 18.2 GHz. Similarly, a sensitivity of 21.75 GHz/RIU with a quality factor of 99.21 was achieved for the refractive index of 1.4.

**Figure 14:**
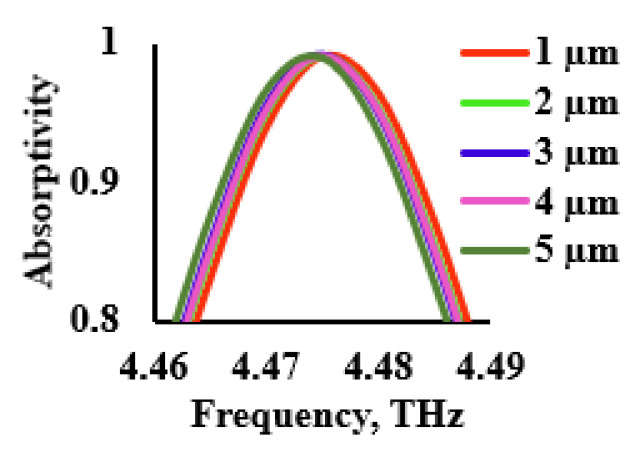
Performance of the sensor for various analyte thicknesses.

The performance analysis of the designed THz sensor was further extended for various thicknesses of the analyte. The analyte thickness was varied from 1 to 5 µm for the refractive index of 1.33 and the respective absorption characteristics were recorded, as shown in Figure 14. From the figure, it can be inferred that, as the thickness of the analyte increased, the redshift was observed. From Figure 14, it was inferred that the increase in the thickness of the analyte caused the redshift of the plasmon resonance due to the change in the effective capacitance of the structure. The average shift in the frequency for varying analyte thickness was estimated to be 1 GHz/µm. 

The performance of the designed graphene-based THz sensor was compared with the existing sensors that have been reported in the literature. This comparison is given in Table 3. It can be inferred from the data that are presented in this table that the proposed THz sensor showed improved sensitivity with maximized miniaturization. From Table 4 it can be inferred that the Q factor of the proposed sensor was higher compared to the Q factor(s) of [30,31] by 78.77%, 55%, 56.4% and 45.2%, respectively.

The salient features of the periodic graphene-based THz sensor are as follows:

- An ultra-miniaturized THz sensor with a footprint of 50 μm × 50 μm was realized. The proposed sensor was 99.98%, 99.82% and 97.95% smaller than the sensors reported in [12,13,14].
- The proposed sensor showed an average sensitivity of 10 GHz/PU greater than 6.94 GHz/PU [33] for the samples of various dielectric material loading.
- The sensor showed an average change in permittivity of 10 GHz/PU for different materials.
- The peak sensitivity of the proposed sensor was 22.75 GHz/RIU at 4.4754 THz, which is greater than the 14.2 GHz/RIU sensitivity level [23] that was realized for the refractive index loading of 1.8.
- The proposed sensor offered a detuning of 1 GHz/µm for samples of different thicknesses. This is a minimal deviation in comparison with the results of the research that was presented in [34].
- Graphene was used in the present research in order to improve the tunability of the proposed sensor, unlike [9] which used gold as the conducting layer.

## 4. Conclusions

A graphene-based THz metamaterial sensor was designed and characterized for various materials based on their refractive indices. The THz sensor consisted of a periodic resonator structure that was made of graphene, which was specifically chosen for its chemical potential tuning characteristics. The results indicate that the proposed sensor offered an average peak absorption of 98.88% at 4.4754 THz. The sensitivity of the sensor for various analytes has been evaluated. It was found that the reported THz sensor had a peak sensitivity of 22.75 GHz/RIU at 4.4754 THz for the refractive index of 1.8. In addition to the sensing performance, the figures of merit for the proposed sensor were analyzed and the results have been presented. From the realized values, it can be concluded that the proposed THz sensor is an optimum solution for material sensing in the THz regime.

## Figures and Tables

**Table 1:** Dimension details of graphene-based THz sensor.

Parameters	L	W	W_1_	W_2_	W_3_	W_4_	G1	G2	t_g_	t_d_	t_c_	t
Value (μm)	50	50	4.9	4.7	2.55	4.9	0.5	0.59	5.2	18	0.25	40

**Table 2:** Absorption characteristics of various refractive indices.

Refractive Index	A (%)	S (GHz/RIU)	Δf (GHz)	FWHM (GHz)	Q	FoM
1	98.88	-	-	50	89.5	-
1.33	98.21	15.15	5	46	97.17	0.33
1.35	97.75	16.57	5.8	45	99.31	0.37
1.38	97.28	19.74	7.5	45	99.28	0.44
1.4	97.28	21.75	8.5	45	99.25	0.48
1.5	97.52	20.8	10.4	45	99.21	0.46
1.8	95.46	22.75	18.2	56	79.58	0.41

**Table 3:** Performance comparison with other relevant THz sensors.

Ref.	Size	*f* (THz)	Sensitivity (GHz/RIU)	% Miniaturization Compared with This Work
[12]	12 mm × 12 mm	0.15	22.2	99.98
[13]	1.2 mm × 1.2 mm	0.1	2.6	99.82
[14]	350 μm × 350 μm	0.508	23.08	97.95
This work	50 μm × 50 μm	4.475	22.75	-

**Table 4:** Comparison of Q factor with other relevant THz graphene-based sensors.

Ref.	Q-Factor	Increased %
[30]	<20	78.77
[31]	40.1	55.19
[32]	<40 at 15.1 THz <50 at 27.2 THz	56.42 45.25
This work	89.5	-
